# The Mitochondrial Fusion-Promoting Factor Mitofusin Is a Substrate of the PINK1/Parkin Pathway

**DOI:** 10.1371/journal.pone.0010054

**Published:** 2010-04-07

**Authors:** Angela C. Poole, Ruth E. Thomas, Selina Yu, Evelyn S. Vincow, Leo Pallanck

**Affiliations:** 1 Department of Genome Sciences, University of Washington, Seattle, Washington, United States of America; 2 Neurobiology and Behavior Program, University of Washington, Seattle, Washington, United States of America; National Institutes of Health, United States of America

## Abstract

Loss-of-function mutations in the *PINK1* or *parkin* genes result in recessive heritable forms of parkinsonism. Genetic studies of Drosophila orthologs of *PINK1* and *parkin* indicate that PINK1, a mitochondrially targeted serine/threonine kinase, acts upstream of Parkin, a cytosolic ubiquitin-protein ligase, to promote mitochondrial fragmentation, although the molecular mechanisms by which the PINK1/Parkin pathway promotes mitochondrial fragmentation are unknown. We tested the hypothesis that PINK1 and Parkin promote mitochondrial fragmentation by targeting core components of the mitochondrial morphogenesis machinery for ubiquitination. We report that the steady-state abundance of the mitochondrial fusion-promoting factor Mitofusin (dMfn) is inversely correlated with the activity of PINK1 and Parkin in Drosophila. We further report that dMfn is ubiquitinated in a PINK1- and Parkin-dependent fashion and that dMfn co-immunoprecipitates with Parkin. By contrast, perturbations of PINK1 or Parkin did not influence the steady-state abundance of the mitochondrial fission-promoting factor Drp1 or the mitochondrial fusion-promoting factor Opa1, or the subcellular distribution of Drp1. Our findings suggest that dMfn is a direct substrate of the PINK1/Parkin pathway and that the mitochondrial morphological alterations and tissue degeneration phenotypes that derive from mutations in *PINK1* and *parkin* result at least in part from reduced ubiquitin-mediated turnover of dMfn.

## Introduction

Parkinson’s disease (PD) is a common neurodegenerative movement disorder caused by the degeneration of dopamine-secreting neurons in the midbrain. The mechanisms underlying dopamine neuron degeneration in PD are incompletely understood, although systemic mitochondrial complex I impairment is a relatively common feature [1], suggesting that mitochondrial dysfunction plays a major role in PD pathogenesis. Further support for this model derives from the findings that particular mitochondrial toxins can elicit PD-like syndromes in humans and animal models [2], [3]; that mitochondrial DNA deletions occur at high frequency in dopaminergic neurons of aged individuals [4], [5], particularly those with PD [4]; and that several genes implicated in familial forms of PD influence mitochondrial integrity in model systems [6], [7], [8].

Recent work in flies and vertebrates suggests that two of the genes involved in familial forms of PD, *PINK1* and *parkin*, act in a common pathway to regulate mitochondrial morphology [6]. *PINK1* encodes a mitochondrially targeted serine/threonine kinase, whereas *parkin* encodes an E3 ubiquitin-protein ligase. In Drosophila, loss-of-function mutations in *PINK1* and *parkin* result in enlarged and swollen mitochondria and degeneration of flight muscle, sperm and dopaminergic neurons [9], [10], [11], [12], [13], [14]. These phenotypes can be at least partially suppressed by increasing the gene dosage of the mitochondrial fission-promoting factor Drp1, or by decreasing the gene dosage of the mitochondrial fusion-promoting factors Opa1 or Mitofusin (dMfn) [15], [16], [17], [18]. These findings suggest that the PINK1/Parkin pathway acts to promote mitochondrial fission or inhibit mitochondrial fusion, either of which would culminate in mitochondrial fragmentation. PINK1 and Parkin have also been shown to influence mitochondrial morphology in vertebrates, although, in contrast to the studies conducted in Drosophila, mitochondrial fragmentation is the most frequently observed phenotype upon reduced PINK1 and Parkin activity in mammalian cell culture [19], [20], [21], [22]. While these discordant findings may reflect differences in the mechanisms by which PINK1 and Parkin influence mitochondrial morphology in vertebrates and invertebrates, this possibility has not yet been tested because the molecular mechanisms by which PINK1 and Parkin influence mitochondrial morphology are unknown.

In our present work, we tested the hypothesis that PINK1 and Parkin influence mitochondrial morphology in Drosophila by acting directly on core components of the mitochondrial fission- or fusion-promoting apparatus. We report that dMfn is ubiquitinated in a PINK1- and Parkin-dependent fashion, that the steady-state abundance of dMfn is inversely correlated to PINK1 and Parkin activity, and that Parkin co-immunoprecipitates with dMfn. Together, our findings suggest that dMfn is a direct substrate of PINK1 and Parkin, and that the effects of reduced PINK1 and Parkin activity on mitochondrial morphology and tissue integrity derive at least in part from reduced ubiquitin-mediated turnover of dMfn.

## Results

**Figure 1:**
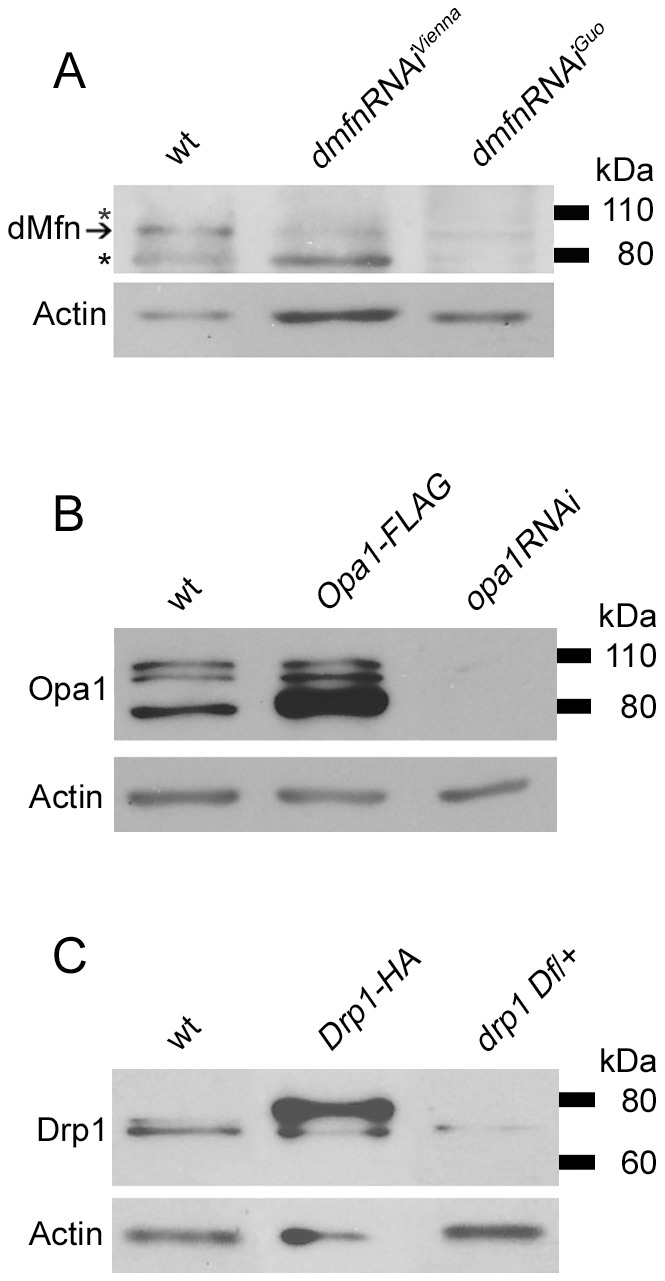
Western blot analysis showing specificity of antisera that recognize Drosophila dMfn, Opa1, and Drp1. (A) Protein extracts from wt flies and flies expressing either the *UAS-dmfn-RNAi^Vienna^* or *UAS-dmfn-RNAi^Guo^* transgenes targeting the *dmfn* gene were subjected to western blot analysis with an anti-dMfn antiserum. The *hsp70-GAL4* driver was used to induce expression of the *dmfn-RNAi* transgenes, and flies were collected for analysis 24 to 48 hrs after a 3-hr heat shock protocol. The arrow indicates the location of the dMfn band at 94 kDa. The asterisks (*) indicate two nonspecific bands detected at 80 kDa and 110 kDa. (B) Protein extracts from wt flies, flies bearing the *UAS-Opa1-FLAG* transgene and the mesoderm-specific *24B-GAL4* driver, and flies bearing the *UAS-opa1RNAi* transgene and the muscle-specific *dmef2-GAL4* driver were subjected to western blot analysis with an anti-Opa1 antiserum. (C) Protein extracts from wt flies, flies bearing the *UAS-Drp1-HA* transgene and the muscle-specific *dmef2-GAL4* driver, and flies heterozygous for the *Df(2L)Excel6008* deletion chromosome (which removes the *drp1* gene) were subjected to western blot analysis with an affinity-purified anti-Drp1 antiserum.

To test the hypothesis that Parkin promotes the ubiquitin-mediated turnover of core components of the mitochondrial morphogenesis machinery, we sought to explore the effects of altered Parkin activity on the steady-state abundance of the mitochondrial fission-promoting factor Drp1 and the mitochondrial fusion-promoting factors Opa1 and dMfn. Although Drosophila encodes a second mitofusin homolog that is expressed exclusively in the male germline [23], [24], we focused on dMfn because of its broad expression pattern [24]. Because PINK1 acts genetically upstream of Parkin [11], [12], [14], we also sought to test whether alterations in PINK1 activity influence the steady-state abundance of Drp1, Opa1 and dMfn. To perform these experiments we generated antisera against Drosophila Drp1 and dMfn using peptide immunogens corresponding to sequences in these proteins. Figure 1 shows the results of western blot analysis using these two affinity-purified antisera, as well as a commercial antiserum that is able to immunoprecipitate Drosophila Opa1 (see Materials and Methods section) to test their specificity.

The anti-dMfn antiserum recognizes several bands, including one of 94 kDa, which corresponds well to the 91–94 kDa sizes of the predicted dMfn isoforms in Drosophila (Figure 1A). The abundance of the 94 kDa band recognized by our antiserum is selectively decreased in flies expressing two different RNAi constructs targeting the *dmfn* transcript, indicating that this band represents dMfn. The other bands detected by our anti-dMfn antiserum are unaffected by RNAi treatment, suggesting that they represent nonspecific cross-reacting species.

The anti-Opa1 antiserum detects bands of 80 kDa, 100 kDa and 105 kDa (Figure 1B). The intensities of all three of these bands are significantly diminished by double-stranded RNAs targeting the *opa1* transcript, and overexpression of a FLAG-tagged Opa1 construct results in the appearance of more intense 80 kDa and 100 kDa bands. In vertebrates, mature Opa1 protein is proteolytically processed into several lower molecular weight forms in the mitochondrion [25]. Thus, the 105 kDa Opa1 band may represent a full-length or near full-length form of Opa1, which is predicted to be between 107 and 112 kDa in Drosophila, whereas the 80 kDa and 100 kDa Opa1 bands may correspond to proteolytically processed forms of this protein. Although we do not understand why Opa1 overexpression selectively influences the abundance of only the lower molecular weight Opa1 species, our findings support the conclusion that this antiserum specifically recognizes Opa1.

The anti-Drp1 antiserum recognizes bands of 70 and 75 kDa in Drosophila, which reasonably approximate the 83 kDa predicted size of this protein (Figure 1C). The intensities of both of these bands are diminished in a fly strain bearing a heterozygous null mutation of *drp1* and increased in transgenic flies overexpressing Drp1, indicating that both bands represent Drp1 and that our antiserum is specific for Drp1. In vertebrates, Drp1 is subjected to multiple posttranslational modifications, including phosphorylation, ubiquitination and sumoylation [26]. Thus, the two different forms of Drosophila Drp1 may derive from a posttranslational modification, or alternatively an uncharacterized splice variant of the *drp1* gene. Together, our findings indicate that our antisera against Drp1, Opa1, and dMfn are able to efficiently recognize these three proteins in Drosophila.

**Figure 2:**
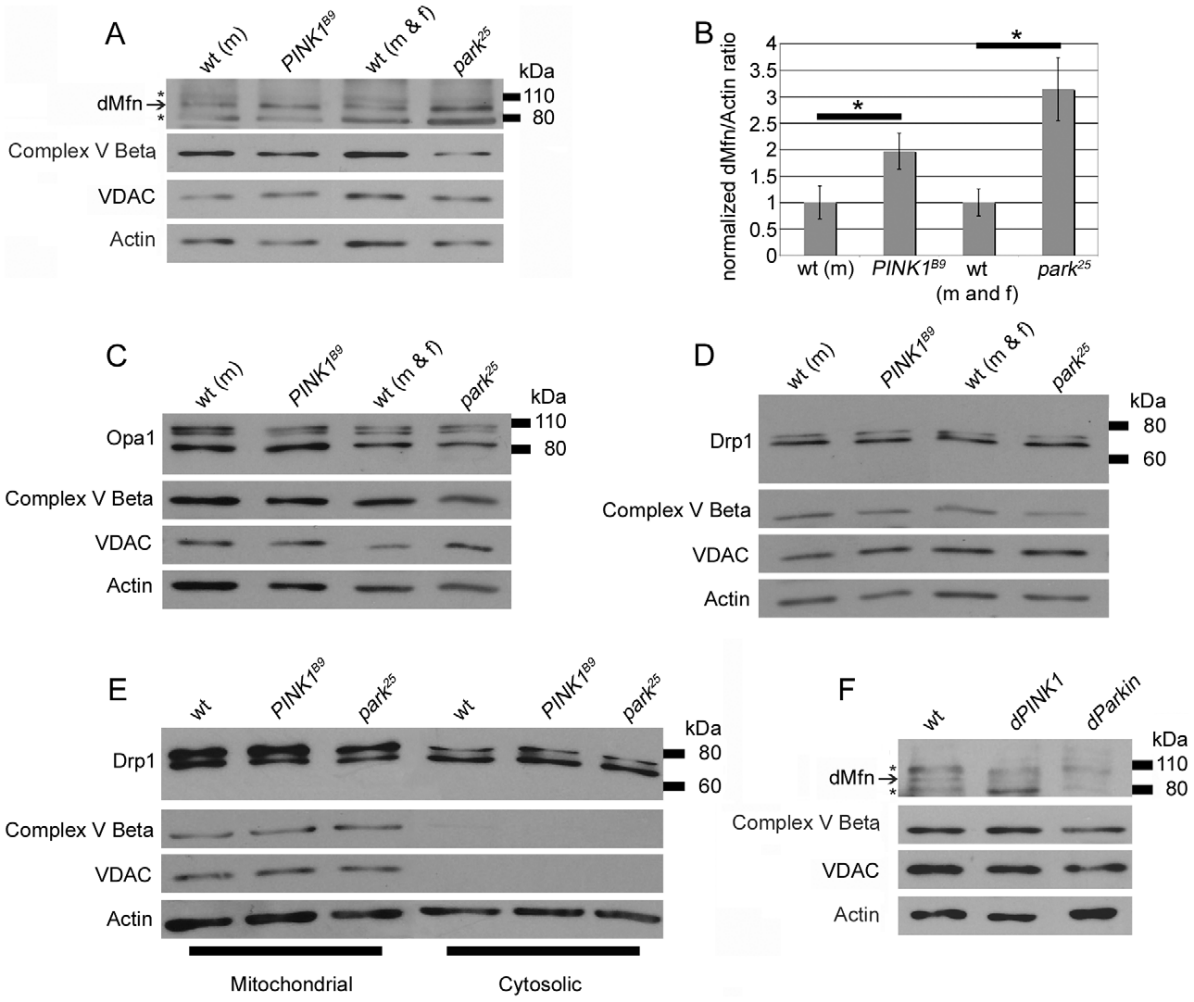
Perturbations of *PINK1* and *parkin* specifically influence dMfn abundance. Protein extracts from wt males (m), *PINK1^B9^* hemizygous males, wt males and females (m and f) and *park^25^* males and females were subjected to western blot analysis with an affinity-purified anti-dMfn antiserum (A), an anti-Opa1 antiserum (C), an affinity-purified anti-Drp1 antiserum (D), an anti-complex V β antiserum (A, C, D), an anti-VDAC antiserum (A, C, D), and an anti-actin antiserum (A, C, D). Arrow in (A) indicates location of the dMfn band at 94 kDa and the asterisks (*) indicate nonspecific bands. (B) Quantification of dMfn abundance in *PINK^B9^* and *park^25^* null mutants relative to wt controls. Because *PINK1^B9^* males are sterile and the *PINK1* gene resides on the X chromosome, we are only able to generate male *PINK1^B9^* mutants and thus used wt males as the control population for these mutants. The ratio of dMfn to actin abundance was obtained from three independent blots for each sample analyzed; the ratios for wt controls were set at a value of 1 and mutant ratios were normalized to the wt ratios. *p<0.05 by Student’s t-test. (E) Protein extracts from mitochondrial and cytosolic fractions (see Materials and Methods section for details on fractionation) were subjected to western blot analysis with an affinity-purified anti-Drp1 antiserum, an anti-complex V β (CompV) antiserum, an anti-VDAC antiserum, and an anti-actin antiserum. (F) Protein extracts from wt flies, flies overexpressing Parkin (*hsp70-GAL4/UAS-Parkin*) and flies overexpressing PINK1 (*hsp70-GAL4/UAS-PINK1*) were subjected to western blot analysis with an affinity-purified anti-dMfn antiserum, an anti-complex V β antiserum, an anti-VDAC antiserum, and an anti-actin antiserum. Flies were subjected to a 1-hr heat shock and collected for analysis 24 hrs following the heat shock. The arrow indicates the location of the dMfn band at 94 kDa and the asterisks (*) indicate the two nonspecific bands.

We used our three antisera to compare the steady-state abundance of Drp1, Opa1, and dMfn in wild-type (wt) flies, *park^25^* null mutants, and *PINK1^B9^* null mutants. These analyses revealed that dMfn abundance was increased in both *park^25^* and *PINK1^B9^* null mutants relative to wt controls (Figure 2A, B). By contrast, we did not detect an alteration in the steady-state abundance or molecular weight of Opa1 or Drp1 in *park^25^* or *PINK1^B9^* null mutants relative to wt controls (Figure 2C, D). Mutations in *PINK1* and *parkin* also failed to influence the molecular weight or abundance of several control proteins that localize to mitochondria, including the β-subunit of mitochondrial complex V and the mitochondrial voltage-dependent anion channel (VDAC, also known as porin), indicating that the increased abundance of dMfn in *PINK1* and *parkin* mutants does not reflect a general increase in mitochondrial mass (Figure 2). There was no significant change in the abundance of the *dmfn* transcript in *park^25^* or *PINK1^B9^* null mutants relative to wt (data not shown), indicating that mutations in *PINK1* and *parkin* influence dMfn protein abundance through a posttranscriptional mechanism.

We also explored the possibility that decreased PINK1 or Parkin activity might influence the subcellular distribution of Drp1, which is recruited to mitochondria from the cytoplasm to promote mitochondrial fission. However, we detected no obvious alteration in the fraction of Drp1 that distributes with mitochondria in *park^25^* or *PINK1^B9^* mutants relative to wt controls (Figure 2E), although this experiment revealed that the higher MW form of Drp1 appeared to preferentially localize to mitochondria in both wt and mutant samples. This finding may offer insight into the mechanism by which Drp1 is recruited to mitochondria and will be further explored in future work.

Given that the abundance of dMfn was increased in *PINK1* and *parkin* mutants, we also tested the effects of PINK1 and Parkin overexpression on dMfn abundance. These studies revealed that overexpression of PINK1 or Parkin resulted in decreased dMfn abundance relative to a wt control (Figure 2F). The effects of PINK1 and Parkin overexpression appeared to be specific to dMfn, as we failed to detect any influence of PINK1 or Parkin overexpression on the abundance or size of the β-subunit of mitochondrial complex V, or of VDAC, which, like dMfn, localizes to the outer mitochondrial membrane (Figure 2F).

**Figure 3:**
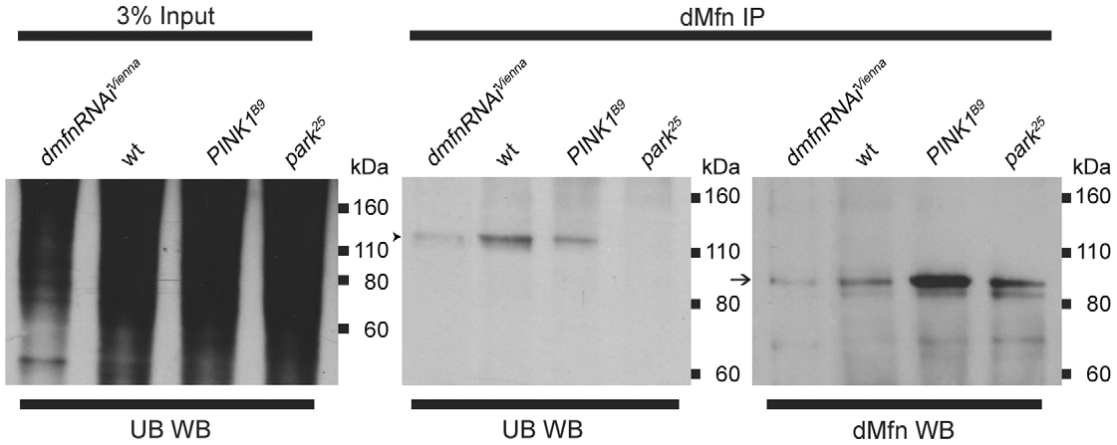
dMfn is ubiquitinated in a PINK1- and Parkin-dependent fashion. Affinity purified anti-dMfn antiserum was used to immunoprecipitate dMfn from wild-type flies, *PINK1^B9^* mutants, and *park^25^* mutants. As a control for specificity, anti-dMfn antiserum was also used to immunoprecipitate dMfn from flies with *hsp70-GAL4* driven expression of *UAS-dmfn-RNAi^Vienna^.* In the left panel, 3% of the lysate input used in the immunoprecipitations was subjected to western blot analysis using an anti-ubiquitin antiserum to show that general ubiquitination levels were similar in all genotypes. In the middle and right panels, dMfn immunoprecipitates from all four genotypes were subjected to western blot analysis with either anti-ubiquitin antiserum (middle panel) or anti-dMfn antiserum (right panel). Arrow indicates the unmodified dMfn species detected in wt flies, *PINK1^B9^* mutants, and *park^25^* mutants, with reduced levels in flies expressing *UAS-dmfn-RNAi^Vienna^*. Arrowhead indicates location of ubiquitinated dMfn species. These analyses were replicated at least three times with similar results.

Our finding that the abundance of dMfn is increased in *PINK1* and *parkin* mutants and decreased upon overexpression of PINK1 and Parkin is consistent with the hypothesis that PINK1 and Parkin promote the ubiquitin-mediated turnover of dMfn. A prediction of this hypothesis is that a ubiquitinated form of dMfn exists, at least transiently, in wt flies, and that the abundance of this ubiquitinated form of dMfn will be substantially decreased in *PINK1* and *parkin* mutants. To test this prediction, we immunoprecipated dMfn from wt flies, *PINK1* mutants and *parkin* mutants and subjected these immunoprecipitates to western blot analysis with an anti-dMfn and an anti-ubiquitin antiserum. To control for the specificity of our anti-dMfn antiserum, we also immunoprecipated dMfn from flies expressing an RNAi construct targeting the *dMfn* transcript and subjected the immunoprecipitate to western blot analysis with an anti-dMfn and an anti-ubiquitin antiserum. The anti-ubiquitin antiserum detected a band of approximately 120 kDa in the immunoprecipitate from wt flies but detected only a very faint 120 kDa band in flies expressing an RNAi construct targeting the *dMfn* transcript (Figure 3), indicating that a low-abundance form of ubiquitinated dMfn exists in wt flies. By contrast, the abundance of ubiquitinated dMfn in the dMfn immunoprecipitates from *PINK1^B9^* null mutants and *park^25^* null mutants was dramatically decreased relative to wt, despite the fact that more dMfn was immunoprecipitated from *PINK1^B9^* and *park^25^* mutants relative to wt (Figure 3). Identical results were obtained upon repeating these experiments using an independently generated anti-dMfn antiserum to immunoprecipitate dMfn from flies of the aforementioned genotypes (Figure S1). Although a small amount of ubiquitinated dMfn is still detectable in *PINK1* null mutants (Figure 3), this finding is consistent with previous genetic studies demonstrating that Parkin is able to compensate at least partially for the loss of PINK1 activity [11], [12], [14], [15]. Moreover, the finding that dMfn is still weakly ubiquitinated in *PINK1* null mutants provides an explanation for the somewhat less severe phenotypes of *PINK1* null mutants relative to *parkin* null mutants [9], [10], [11], [12], [13], [15].

**Figure 4:**
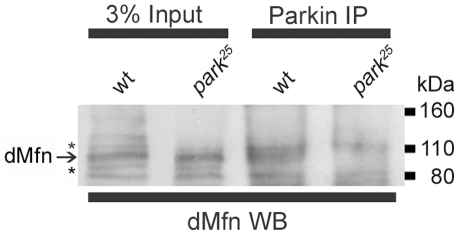
dMfn assembles with Parkin. Affinity purified anti-Parkin antiserum was used to immunoprecipitate Parkin from wt flies and *park^25^* null mutants. As *park^25^* flies do not produce any Parkin protein, this immunoprecipitation serves as a control for any nonspecific binding of other proteins to the anti-Parkin antiserum. Three percent of the total amount of lysate used for the immunoprecipitations as well as the Parkin immunoprecipitates were subjected to western blot analysis with the anti-dMfn antiserum. Only in the wt flies did a band corresponding to the dMfn band appear indicating an interaction between Parkin and dMfn. The asterisks (*) indicate the nonspecific bands.

Our findings are consistent with the model that the PINK1/Parkin pathway promotes the ubiquitin-mediated turnover of dMfn. While the simplest interpretation of our findings is that Parkin directly promotes the ubiquitination of dMfn, we cannot rule out the possibility that the ubiquitination of dMfn by Parkin proceeds through an indirect mechanism. To help distinguish between these models we immunoprecipitated Parkin from wt flies and subjected the immunoprecipitate to western blot analysis with an anti-dMfn antiserum. To control for the possible nonspecific interaction of dMfn with our anti-Parkin antiserum, we also subjected a protein extract from *park^25^* null mutants to immunoprecipitation with an anti-Parkin antiserum and then used this immunoprecipitate in a western blot analysis with our anti-dMfn antiserum. These experiments revealed that dMfn co-immunoprecipated with Parkin in wt flies, but failed to detect a dMfn band in an immunoprecipitate from *park^25^* mutants (Figure 4). These findings indicate that Parkin assembles in a complex with dMfn and suggest that dMfn is a direct substrate of Parkin.

## Discussion

**Figure 5:**
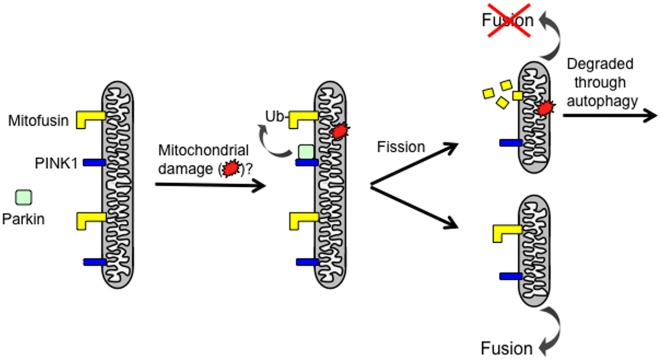
A potential model by which the PINK1/Parkin pathway promotes mitochondrial fragmentation and turnover. Previous work has shown that PINK1 localizes to the outer mitochondrial membrane with its kinase domain facing the cytoplasm [28], [39], where it is required to recruit Parkin selectively to damaged mitochondria to promote the autophagic clearance of these mitochondria [27], [28], [29], [30], [31]. Our model postulates that upon Parkin recruitment to damaged portions of the mitochondrial reticulum, it ubiquitinates (Ub) the mitochondrial fusion-promoting factor Mitofusin, thus tagging it for degradation, or otherwise inactivating its fusion-promoting activity. Subsequent Drp1-dependent mitochondrial fission would yield a damaged mitochondrial product that is deficient in Mitofusin, and thus unable to re-enter the mitochondrial network. These small damaged mitochondrial fission products would instead be targeted for autophagic degradation. Although our figure depicts a model in which Parkin binds prior to mitochondrial fission, Parkin could also be recruited to damaged mitochondria following fission. An alternative model is that the ubiquitination of Mitofusin serves as a signal for the turnover of damaged mitochondria. These possible models are not mutually exclusive.

In previous work, we and others showed that genetic manipulations that promote mitochondrial fragmentation, including increased *drp1* gene dosage and decreased *opa1* or *dmfn* gene dosage, dramatically suppress the *PINK1* and *parkin* mutant phenotypes in Drosophila [15], [16], [17], [18]. These findings, coupled with previous work demonstrating that PINK1 acts upstream of Parkin in a common pathway [11], [12], [14], led us to hypothesize that PINK1 and Parkin influence mitochondrial integrity by regulating core components of the mitochondrial morphogenesis machinery through ubiquitination [15]. Our current results provide direct support for this hypothesis by demonstrating that dMfn is ubiquitinated in a PINK1- and Parkin-dependent fashion and that the steady-state abundance of dMfn is increased in *PINK1* and *parkin* mutants and decreased in PINK1- and Parkin-overexpressing flies. These findings suggest a model in which PINK1 phosphorylates either dMfn or Parkin and thereby increases the efficiency with which Parkin is able to ubiquitinate dMfn. The subsequent ubiquitin-mediated turnover of dMfn would then inhibit mitochondrial fusion, and thus promote mitochondrial fragmentation (Figure 5). While our current findings were in review, another study primarily using cultured Drosophila S2 cells also reported that dMfn is a substrate of the PINK1/Parkin pathway, thus providing additional support for our conclusions [27].

Our previous finding that the PINK1/Parkin pathway promotes mitochondrial fragmentation led us to propose that this pathway may act to segregate damaged portions of the mitochondrial reticulum for turnover through an autophagic mechanism [15]. Several recent studies provide compelling support for this hypothesis by demonstrating that treatment of cultured vertebrate cells with mitochondrial damaging agents triggers PINK1 to selectively recruit Parkin to damaged mitochondria, where Parkin acts to promote the autophagic turnover of these mitochondria, presumably by ubiquitinating particular mitochondrial targets [28], [29], [30], [31]. These studies, together with our current findings raise the possibility that the selective Parkin-mediated ubiquitination and subsequent degradation of dMfn on damaged portions of the mitochondrial reticulum, coupled with ongoing mitochondrial fission serves to sequester the mitochondrial damage to small fusion-incompetent mitochondria that are subsequently eliminated through autophagy (Figure 5). However, the size of ubiquitinated dMfn suggests that it is triply ubiquitinated and previous work indicates that a chain of four or more ubiquitins is required for efficient targeting to the proteasome [32]. Thus, alternative interpretations of our findings, although not mutually exclusive, are that ubiquitination of dMfn inactivates the fusion-promoting activity of dMfn, or serves as a tag marking the damaged mitochondria for destruction by autophagy. The finding that the ubiquitination of a peroxisomal surface protein is sufficient to signal the autophagic degradation of this organelle [33] is consistent with our latter model. Experiments are currently underway to distinguish these possibilities.

While our model in which the PINK1/Parkin pathway promotes mitochondrial fragmentation through the ubiquitination of dMfn is completely consistent with previous work on PINK1 and Parkin in Drosophila, recent findings from vertebrate cell culture studies challenge this model. In particular, several of the studies of PINK1 in vertebrate systems have found that reduced PINK1 activity results in mitochondrial fragmentation [19], [20], [21], [22], suggesting that PINK1 may promote mitochondrial fusion—exactly the opposite of the conclusion drawn from studies of the PINK1/Parkin pathway in flies. While additional work will be required to resolve these apparent conflicts, it is important to point out that the findings from studies of PINK1 and Parkin in flies have involved tissues that are profoundly affected by loss of PINK1 and Parkin activity, whereas the tissue sources of the cells that have been used in at least some of the conflicting vertebrate studies are largely unaffected by mutations in *PINK1* and *parkin*. Thus, a possible explanation for these apparently discordant findings is that the mitochondrial fragmentation resulting from reduced PINK1 activity that has been observed in vertebrate systems involves a compensatory induction of mitochondrial fragmentation in these cells, which perhaps also explains their relative insensitivity to the loss of PINK1 activity. In potential support of this model is the finding that while the mitochondrial fragmentation seen in PINK1-deficient vertebrate cells can be rescued by inactivating Drp1, this manipulation enhances the cell death associated with PINK1 deficiency [21], a finding that is entirely consistent with work in flies. Future work should resolve these apparent conflicts and further clarify the influence of PINK1- and Parkin-dependent ubiquitination of dMfn on mitochondrial integrity.

## Materials and Methods

### 

#### Fly strains

The *UAS-PINK1* [12], *UAS-Park* [9], *UAS-Opa1-FLAG* [16], *UAS-Drp1-HA* [16], *UAS-opa1-RNAi* [17], *UAS-dmfn-RNAi^Guo^* [17], *dmef2-GAL4* [34], *elav-GAL4* [35], *24B-GAL4* [36] and *hsp70-GAL4* [37] transgenic lines and the *park^25^* [9] and *PINK1^B9^* [12] alleles have all been described previously. A UAS-regulated RNAi line targeting the *dmfn* transcript (*UAS-dmfn-RNAi^Vienna^*) was obtained from the Vienna Drosophila RNAi Center (Vienna, Austria). The *Df(2L)Excel6008* deletion stock, which removes the *drp1* gene, was obtained from the Bloomington Stock Center (Bloomington, IN).

#### Heat shock induction protocols

Flies bearing both the *hsp70-GAL4* driver and the GAL4-responsive transgenes of interest were subjected to heat shock by incubation for 1 hr at 37°C. For the RNAi transgenes, the flies were incubated a further 2 hrs at 30°C. Following heat shock, flies were either flash frozen in liquid nitrogen, or maintained at 18°C for varying periods of time before freezing.

#### Antisera

Rabbit polyclonal antiserum recognizing Drosophila Parkin has been previously described [38]. Rabbit polyclonal antisera recognizing the Drosophila Drp1 and dMfn were generated by a commercial source (Pocono Rabbit Farm, Canadensis, PA) using synthetic peptides corresponding to sequences in these proteins (Drp1: PPAPTRPDSIENST; dMfn: FTGKVRERSKKGQP). The anti-Drp1 and anti-dMfn antisera were purified using the synthetic peptides that elicited these antisera as ligands in affinity chromatography, as described (ThermoScientific SulfoLink Immobilization Kit for Peptides, Thermo Scientific (Waltham, MA) #44999). In the course of testing whether a commercial rabbit antiserum to human Mitofusin 2 (Sigma (St. Louis, MO) #M6319) would cross-react with the corresponding fly protein, this antiserum was used to immunoprecipitate proteins from an adult fly protein extract and the immunoprecipitate was subjected to tandem mass spectrometry analysis. This analysis detected 11 Opa1 peptides (13.7% sequence coverage), but failed to detect peptides corresponding to either of the two fly Mitofusin proteins. Additional evidence in support of the specificity of this antiserum for Opa1 is provided in Figure 1. A mouse monoclonal anti-ubiquitin antiserum (Ub (P4D1), sc-8017; Santa Cruz Biotechnology, Santa Cruz, CA) was used to detect ubiquitinated proteins. Mouse monoclonal antibodies were used to detect the inner mitochondrial membrane protein OxPhos Complex V subunit β (Molecular Probes (Eugene, OR) #A 21351), the outer mitochondrial membrane protein porin (VDAC) (MitoSciences (Eugene, OR) #MSA03) and actin (Chemicon (Billerica, MA) #MAB1501).

#### Western blot analyses

Proteins were separated by SDS-PAGE on either 10% Tris acrylamide gels or NuPAGE 4–12% Bis-Tris gels (Invitrogen #NP0335) and electrophoretically transferred onto nitrocellulose or PVDF membranes. Immunodetections with commercial antibodies were performed at the following concentrations: rabbit anti-hMfn2 (dOpa1) 1∶500, mouse anti-OxPhos Complex V subunit β 1∶20,000, mouse anti-VDAC 1∶1000, mouse anti-actin 1∶50,000, mouse anti-ubiquitin 1∶500. The secondary antibodies, anti-rabbit HRP and anti-mouse HRP (Sigma), were used at 1∶10,000 and detection of signal utilized Thermo Scientific electrochemiluminescence (ECL) reagents. Quantification was performed using the software program NIH Image 1.62 (National Institutes of Health, Bethesda, MD) by measuring band density and subtracting background from an identical-sized region of the scanned image lacking bands.

#### Immunoprecipitations

To generate protein extracts for immunoprecipitation, 100–200 adult flies were frozen in liquid nitrogen, ground to a fine powder using a chilled mortar and pestle, transferred to a Dounce homogenizer and homogenized in 1.5 ml of lysis buffer (50 mM Tris-HCl (pH 7.4), 150 mM NaCl, 1% NP-40, 10% glycerol, 10 mM NaF, 1 mM Na_3_VO_4_, 100 µg/ml PMSF, Sigma protease inhibitor cocktail (Sigma #P8340)). The resulting lysate was subjected to centrifugation at 12,000 *g* for 15 minutes at 4°C to remove cuticular material and cell debris. The protein concentration of the supernatant was adjusted to 2 µg/µl with lysis buffer and incubated with purified dMfn antisera overnight at 4°C. Protein G Sepharose (GE Healthcare, Piscataway, NJ) beads were then added to the lysate for 3 hours, collected by low-speed centrifugation, washed three times in lysis buffer and boiled in 2X SDS-PAGE sample buffer with 2% beta-mercaptoethanol for 10 minutes to release immunoprecipitated dMfn. The boiled sample was then subjected to centrifugation to pellet the beads and the supernatant was analyzed using western blot analysis.

#### Subcellular fractionation

Fifty adult flies were manually homogenized in a microfuge tube with a small pestle in subcellular fractionation buffer (220 mM mannitol, 68 mM sucrose, 20 mM HEPES (pH 7.4), 80 mM KCl, 0.5 mM EGTA, 2 mM Mg(CH_3_COO)_2_, and Sigma protease inhibitor cocktail (Sigma #P8340)). The homogenate was then transferred to a Dounce homogenizer and further homogenized with 30 strokes of the pestle, and then subjected to centrifugation at 1,500 *g* for 5 minutes at 4°C to prepare a post-nuclear supernatant. The post-nuclear supernatant was subjected to centrifugation at 10,000 *g* for 25 minutes at 4°C to pellet mitochondria and yield a post-mitochondrial supernatant consisting of soluble cytosolic proteins. The post-mitochondrial supernatant and mitochondrial pellet were then suspended in SDS-PAGE sample buffer and used in western blot analysis.

## Supporting Information

Figure S1 An independently generated anti-dMfn antiserum confirms that dMfn is ubiquitinated in a PINK1- and Parkin-dependent fashion. An anti-dMfn antiserum generated against the peptide DTVDKSGPGSPLSRF was provided by Dr. Alex Whitworth and used to immunoprecipitate dMfn from wt flies, PINK1[B9] mutants, and park[25] mutants. Lysate from flies with hsp70-GAL4 driven expression of UAS-dmfn-RNAi[Vienna] was also subjected to immunoprecipitation to confirm the specificity of the anti-dMfn antiserum provided by Dr. Whitworth. In the left panel, 3% of the lysate input used in the immunoprecipitations was subjected to western blot analysis using an anti-ubiquitin antiserum to show that general ubiquitination levels were similar in all genotypes. In the middle and right panels, the dMfn immunoprecipitates derived using the anti-dMfn antiserum provided by Dr. Whitworth were subjected to western blot analysis with either anti-ubiquitin antiserum (middle panel) or our anti-dMfn antiserum (right panel). Arrow indicates the unmodified dMfn species detected in wt flies, PINK1[B9] mutants, and park[25] mutants, with reduced levels in flies expressing UAS-dmfn-RNAi[Vienna]. Arrowhead indicates location of ubiquitinated dMfn species. All analyses shown were repeated twice with similar results.
(1.31 MB TIF)
