# Supplementary figures and images for: The Mitochondrial Fusion-Promoting Factor Mitofusin Is a Substrate of the PINK1/Parkin Pathway
Source: PLoS One. 2010 Apr 7;5(4):e10054. doi: 10.1371/journal.pone.0010054 (PMC2850930; doi:10.1371/journal.pone.0010054)

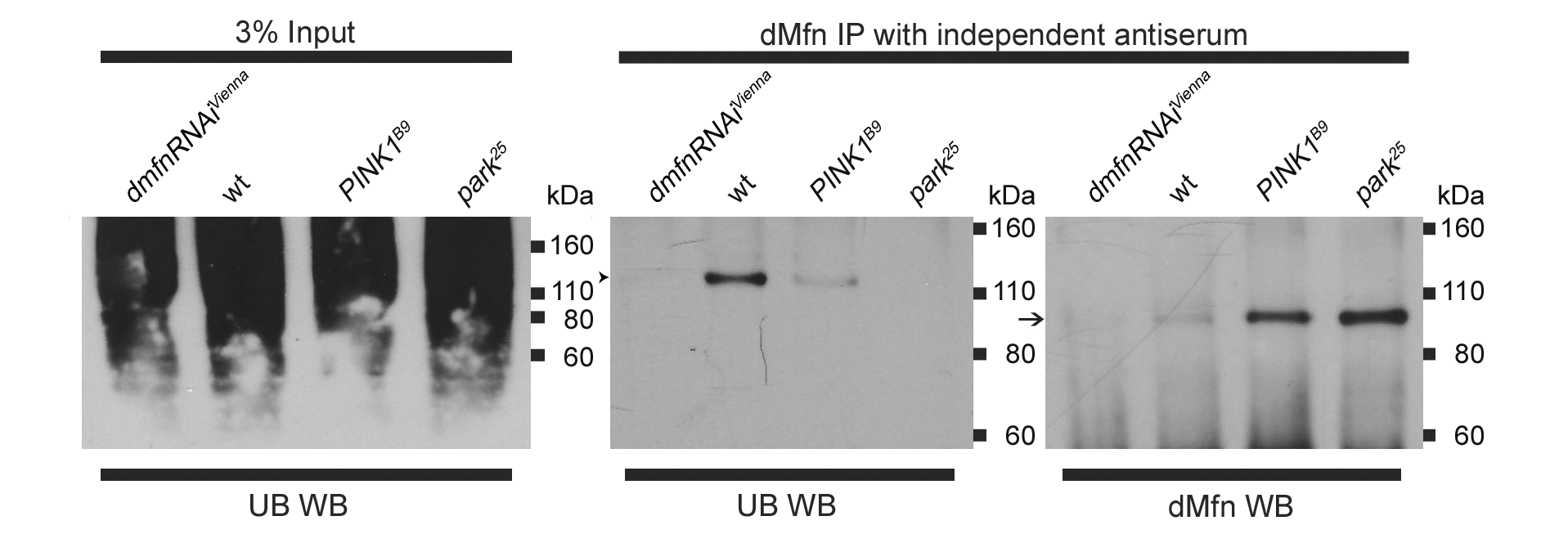

Supplement: Figure S1 — An independently generated anti-dMfn antiserum confirms that dMfn is ubiquitinated in a PINK1- and Parkin-dependent fashion. An anti-dMfn antiserum generated against the peptide DTVDKSGPGSPLSRF was provided by Dr. Alex Whitworth and used to immunoprecipitate dMfn from wt flies, PINK1[B9] mutants, and park[25] mutants. Lysate from flies with hsp70-GAL4 driven expression of UAS-dmfn-RNAi[Vienna] was also subjected to immunoprecipitation to confirm the specificity of the anti-dMfn antiserum provided by Dr. Whitworth. In the left panel, 3% of the lysate input used in the immunoprecipitations was subjected to western blot analysis using an anti-ubiquitin antiserum to show that general ubiquitination levels were similar in all genotypes. In the middle and right panels, the dMfn immunoprecipitates derived using the anti-dMfn antiserum provided by Dr. Whitworth were subjected to western blot analysis with either anti-ubiquitin antiserum (middle panel) or our anti-dMfn antiserum (right panel). Arrow indicates the unmodified dMfn species detected in wt flies, PINK1[B9] mutants, and park[25] mutants, with reduced levels in flies expressing UAS-dmfn-RNAi[Vienna]. Arrowhead indicates location of ubiquitinated dMfn species. All analyses shown were repeated twice with similar results. (1.31 MB TIF) [file pone.0010054.s001.tif]
